# Performance Optimization of Shield Muck-Based Grouting Materials Using Rice Husk Ash and Micro-Expansive Agents

**DOI:** 10.3390/ma19163528

**Published:** 2026-08-20

**Authors:** Jianwei Fan, Shihao Dai, Fang Hu, Kaiwei Liu, Ke Li, Juntao Zhu

**Affiliations:** 1 School of Civil Engineering, Zhengzhou University, Zhengzhou 450001, Chinairwinlike@163.com (K.L.); 2 School of Civil Engineering, Henan Vocational College of Water Conservancy and Environment, Zhengzhou 450008, China; 3 China Railway No. 10 Engineering Group Co., Ltd., Jinan 250101, China; 4 State Key Laboratory of Tunnel Boring Machine and Intelligent Operations, Zhengzhou 450001, China

**Keywords:** waste utilization, shield muck, rice husk ash, grouting materials, early-age performance

## Abstract

Reusing shield muck in grouting materials offers a sustainable approach to reducing waste from underground construction. However, shield muck-based grouts often exhibit poor workability, early-age shrinkage, and insufficient mechanical performance at high water-to-binder ratios. This study investigated the effects of rice husk ash (RHA) and micro-expansive agents on the workability, early-age deformation, mechanical properties, and hydration characteristics of shield muck-based grouting materials. The influences of RHA substitution rate, water-to-binder ratio, muck particle size, and expansive agent type and dosage were evaluated through macroscopic and microstructural tests. The incorporation of RHA reduced the consistency value and setting time by 12.5–27.5% and 31.5–39.9%, respectively. The combined use of RHA and a micro-expansive agent further improved early-age volumetric stability. Compared with the unmodified reference mixture R0W2.4M, the volume shrinkage ratio of the modified mixture was reduced by 78.2%. The mechanical performance depended on the RHA substitution rate and expansive agent dosage. Among the investigated mixtures, the mixture with a water-to-binder ratio of 2.6, 25% RHA, large particle size shield muck, and an appropriate expansive agent dosage showed favorable overall performance. The results demonstrate that RHA and micro-expansive agents can effectively mitigate shrinkage and improve the engineering performance of shield muck-based grouting materials.

## 1. Introduction

Metropolitan infrastructures have experienced rapid development with the acceleration of urbanization. Owing to the higher operating efficiency, energy conservation, and spatial utilization, metro systems have been widely developed around the world, especially in China [1]. From December 2015 to August 2025, the total operating mileage of urban rail transit in China increased to approximately 2.10 times its 2015 level [2]. However, the quick development of the metro may cause some new engineering problems. Among them, the most noticeable concern is that a large amount of shield muck is produced during the shield tunnel construction process [3], which can significantly increase construction expenses and contaminate the environment [4,5,6]. In China, the construction of the metro generates more than 50 million m^3^ of shield muck per year [7], with a disposal cost of over 350 CNY/m^3^ [8]. As a result, in order to lower metro construction costs while also protecting the environment, reliable procedures for disposing of waste shield muck must be provided.

The reutilization of shield muck in the preparation of synchronous grouting materials not only enables the recycling of excavated waste but also reduces environmental pollution and construction costs [9]. The physical properties and phase composition of shield muck generally satisfy the performance requirements of bentonite and fine sand used in conventional grouting materials [10]. Through the incorporation of suitable admixtures and optimization of the mix proportions, the modified grout can achieve well-graded particles and improved mechanical stability, along with desirable properties such as appropriate apparent density, consistency, setting time, permeability, stone rate, compressive strength, and water-soil strength ratio [9,11]. However, excessive shrinkage of cement-based slurry may cause voids to form behind the shield segments [12]. To mitigate this issue, expansive agents are commonly added to adjust the volume stability of the grout. When high soundless cracking agent (HSCA) is employed, the hardened slurry undergoes rapid post-solidification expansion, generating significant expansion pressure [13] that effectively compensates for shrinkage induced by water loss. Nevertheless, the overall reutilization efficiency of shield muck remains low, as only a small fraction of the excavated material is currently recycled, highlighting the urgent need for cementitious system modification strategies to enhance its incorporation rate.

Rice husk ash (RHA) is the byproduct of burning rice husks [14]. RHA is a promising material for partial cement substitution and improving the engineering properties of cement-based materials, owing to its greater SiO_2_ content, significant pozzolanic activity, and higher porosity [15,16,17,18]. The incorporation of RHA can accelerate certain hydration processes, reduce cumulative hydration heat, and modify setting behavior [19], the autogenous and drying shrinkage of cement-based materials decreases by approximately 23.8–41.6% after utilizing the RHA partial substitution of cement, owing to the absorption and desorption capacity of the RHA [20,21]. Additionally, the mechanical strength of cement-based materials increases almost after RHA replace 10 wt%–30 wt% of cement, attributing to the pozzolanic reaction of RHA [22,23,24]. Based on the above findings, the partial replacement of cement with RHA may improve the engineering performance of cement-based grouting materials. However, the combined effects of RHA and expansive agents on the shrinkage behavior, mechanical properties, and microstructural development of shield muck-based grouting materials have not yet been sufficiently investigated.

Previous studies have demonstrated the potential of RHA to modify the shrinkage and mechanical properties of conventional cement-based materials. However, its effects on the workability, volumetric stability, and mechanical performance of shield muck-based grouting materials remain insufficiently understood, particularly when RHA is combined with expansive agents. Therefore, the main objective of this study was to clarify the individual and combined effects of RHA and expansive agents on the engineering performance of shield muck-based grouting materials. Specifically, the effects of the RHA substitution rate, water-to-binder ratio, and shield muck particle size on consistency, setting time, bleeding, early-age deformation, and compressive strength were evaluated. The effects of the type and dosage of expansive agents on the volumetric deformation and mechanical performance of RHA-modified mixtures were then investigated. Finally, X-ray diffraction and scanning electron microscopy analyses were conducted to relate the hydration products and microstructural characteristics to the observed macroscopic performance. This study provides a basis for the effective reuse of shield muck and RHA in grouting materials.

## 2. Experiment

### 2.1. Raw Materials

**Figure 1:**
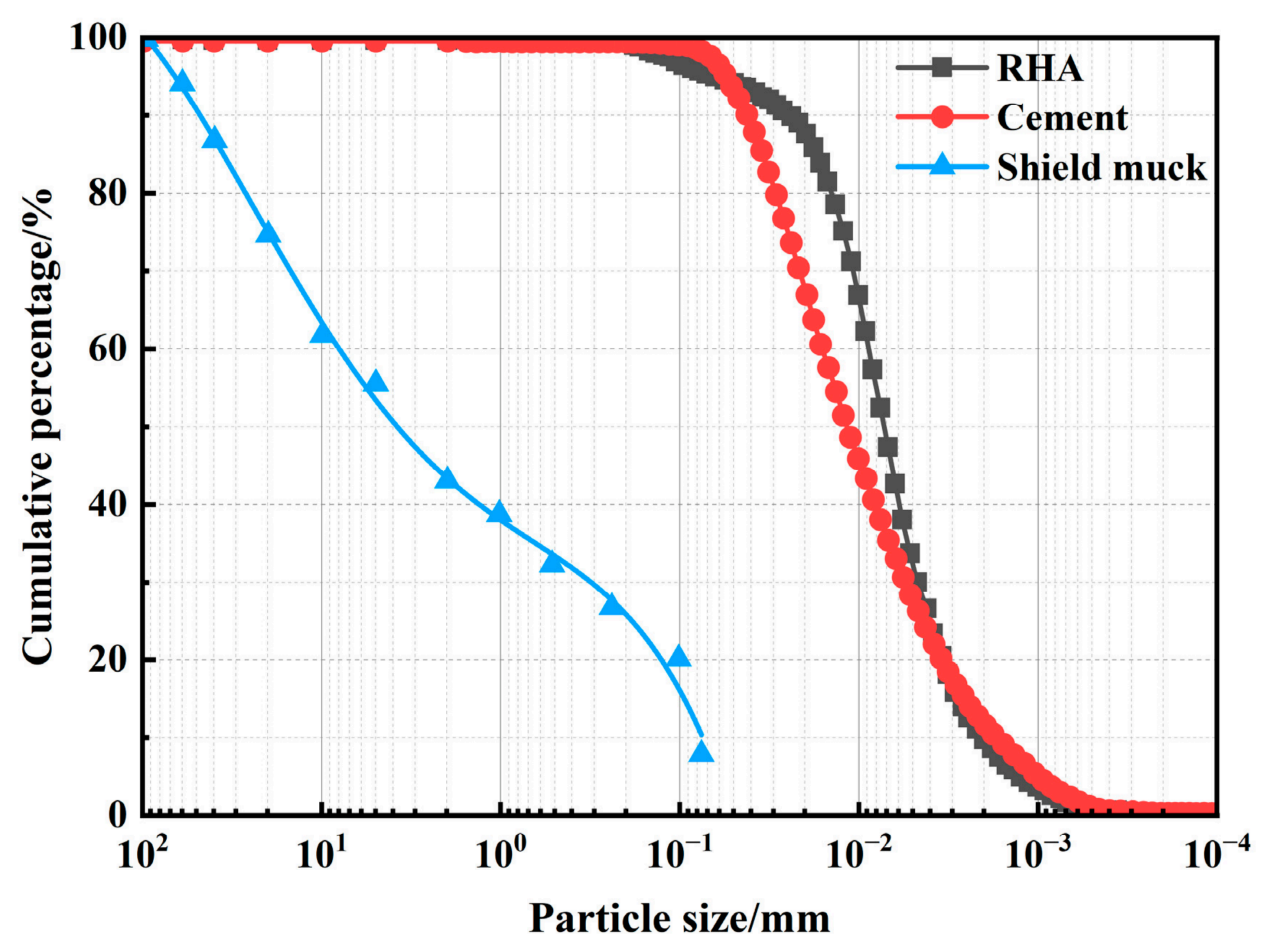
Particle gradation curve of cement, shield muck and RHA.

**Figure 2:**
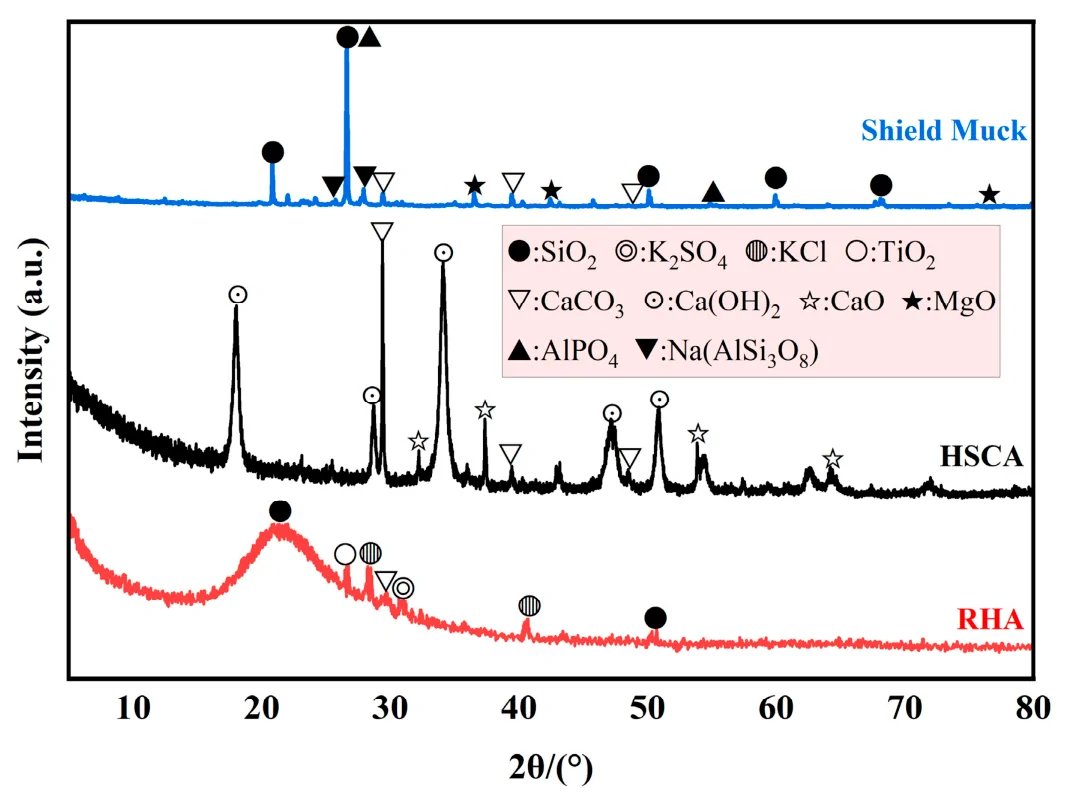
XRD pattern and mineral composition of RHA, HSCA and shield muck.

**Figure 3:**
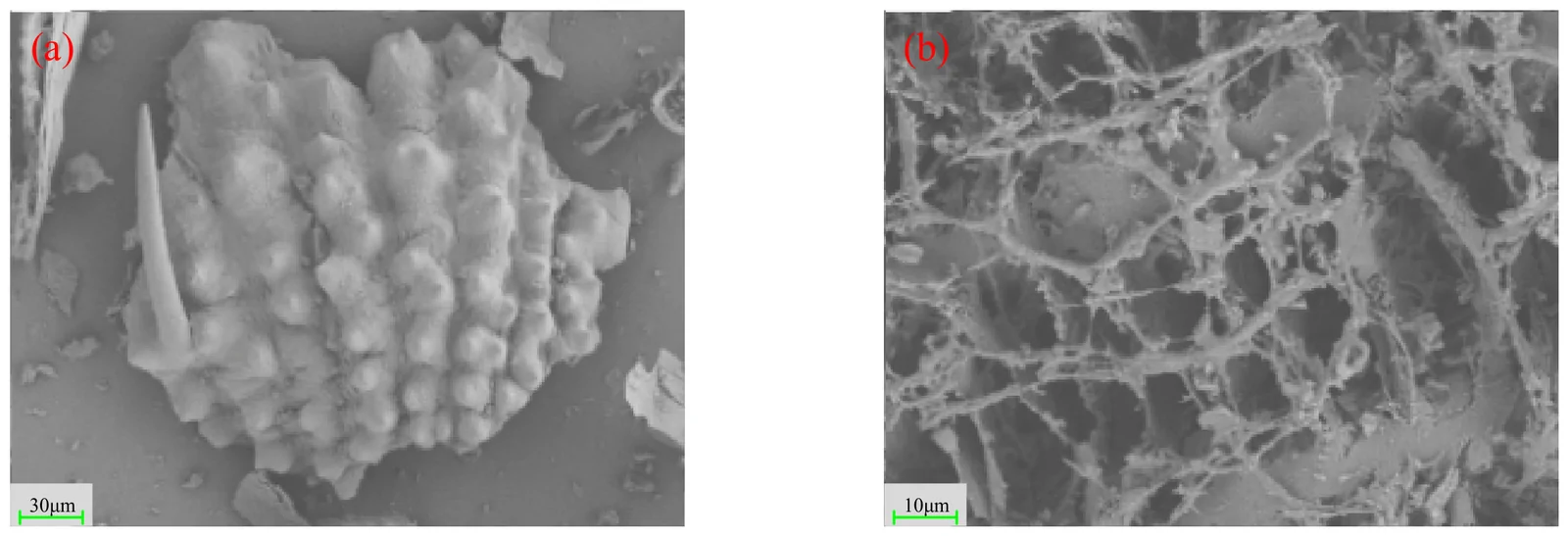
SEM images of rice husk ash: (**a**) overall particle morphology; (**b**) porous honeycomb-like microstructure at a higher observation scale.

The raw materials used in this study mainly include cement, shield muck, RHA, water reducer, expansive agent, and water. P.O. 42.5 cement (similar to ASTM C150 [25]) produced by Tianrui Group Zhengzhou Cement Co., Ltd. (Zhengzhou, China), was utilized as the binder. Its basic properties are presented in Table 1. The shield muck was excavated from the Zhengzhou Metro project, with a moisture content of 15.5% and a density of 1250 kg/m^3^. The liquid limit, plastic limit, and plasticity index of the shield muck were not measured in the present study. Impurities were removed and the soil was dried before testing. According to the particle size distribution curve (Figure 1), the soil is classified as gravel with fine-grained soil in engineering classification. The XRD pattern (Figure 2) shows that the main mineral component of the muck is quartz, with small amounts of periclase, montmorillonite, berlinite, albite, calcite, and other minerals. The RHA used was a commercial product produced by Xiamen C.Miners Technology Co., Ltd. (Xiamen, China), appearing as a blackish-gray powder. According to the XRD pattern (Figure 2) of RHA, its main component is SiO_2_, with small amounts of TiO_2_, K_2_SO_4_, KCl, CaCO_3_, and others. The SEM observation results of RHA are shown in Figure 3a,b. A large number of spherical particles with sizes ranging from 20 to 30 μm can be clearly observed in Figure 3a, agglomerated together, indicating that the RHA was sufficiently combusted. In Figure 3b, numerous micron-scale (0–10 μm) honeycomb pores are regularly arranged, formed by the interlacing of rice husk fiber sheets, which endows RHA with a large specific surface area and high porosity. A polycarboxylate superplasticizer (PC) with a water reduction rate of 25% was used. Two types of expansive agents were adopted, namely quicklime powder with a CaO purity of approximately 90% and high soundless cracking agent (HSCA). HSCA appears as a grayish-white powder. As can be seen from the XRD pattern (Figure 2), its main components include overburned lime (the main expansion source), CaCO_3_, and Ca(OH)_2_. In addition, it contains sodium-based bentonite (as an expansion-enhancing agent), retarders such as sugars and alcohols, as well as hydraulic materials including cement and gypsum.

### 2.2. Mix Proportion Design

Based on the synchronous grouting mixture used in the Zhengzhou Metro project [26] and the results of preliminary screening tests, the binder-to-soil ratio was fixed at 0.20. In this study, the binder-to-soil ratio was defined as the mass ratio of the total binder, including cement and RHA, to dry shield muck. The preliminary tests indicated that a binder-to-soil ratio of 0.20 provided a stable base mixture with acceptable workability, bleeding behavior, setting characteristics, and compressive strength while maintaining a relatively high shield muck content. Therefore, this ratio was adopted as the baseline value and kept constant throughout the experimental program to isolate the effects of the RHA substitution rate, water-to-binder ratio, and shield muck particle size.

The effects of shield muck particle size (0–9.5 mm), water-to-binder ratio (2.4–2.6), and RHA substitution rate (0–25%) on the performance of the base mixtures were subsequently investigated. A polycarboxylate superplasticizer was added to all mixtures at 0.6% by mass of the total binder to regulate workability. Based on the macroscopic performance of the R25W2.6L mixture and the results of preliminary screening tests, quicklime and HSCA were separately incorporated at dosages of 3%, 4%, 5%, 6%, and 7% to systematically evaluate their dosage-dependent effects on workability, early-age deformation, and compressive strength. The expansive agent dosage was defined as the mass of the expansive agent divided by the total mass of water, cement, RHA, and shield muck. The detailed mix proportions are presented in Table 2 and Table 3.

### 2.3. Sample Preparation

**Figure 4:**
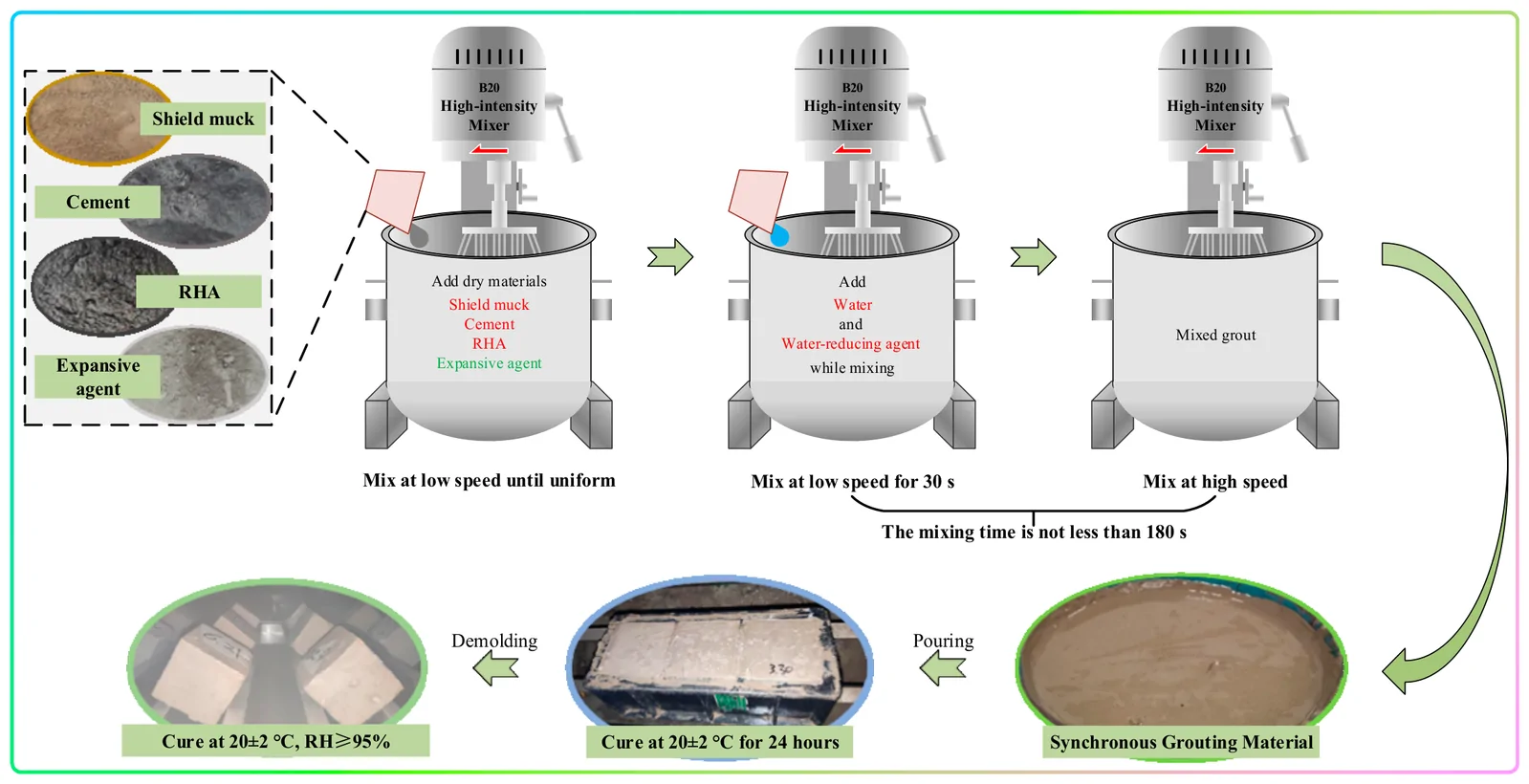
Preparation procedure of shield muck-based grouting specimens.

The slurry preparation and mixing were carried out using a B20 high-intensity mixer (Guangdong Lifeng Machinery Manufacturing Co., Ltd., Zhaoqing, China), as shown in Figure 4. Prior to mixing, both the mixing barrel and the stirring paddles were thoroughly moistened. According to the designed mix proportions, all required materials were weighed sequentially. The dry components, namely shield muck, cement, rice husk ash, and expansive agent, were first added into the mixing barrel and blended at low speed until a homogeneous mixture was obtained. Subsequently, part of the mixing water was used to dilute the water-reducing agent, after which the diluted solution and the remaining water were gradually introduced during continuous mixing. The mixture was stirred at low speed for 30 s, followed by high-speed mixing, ensuring that the total mixing duration after water addition was no less than 180 s.

The prepared grout was cast into cubic molds with dimensions of 70.7 mm × 70.7 mm × 70.7 mm and manually compacted to ensure adequate consolidation. The surface was then leveled with a straight edge to remove excess material. The specimens were kept undisturbed for 24 h at 20 ± 2 °C before demolding, and subsequently transferred to a standard curing room for further conditioning. Daily measurements of linear expansion were performed, and compressive strength tests were conducted after curing periods of 3 and 28 days.

### 2.4. Test Methods

The test methods and their corresponding normative references are summarized in Table 4. The Chinese standards listed in the table were followed in the experimental procedures, while the relevant international standards are provided for methodological reference and comparison.

Experimental data were plotted using OriginPro 2024 (OriginLab Corporation, Northampton, MA, USA), and schematic diagrams were prepared using Microsoft Visio 2013 (Microsoft Corporation, Redmond, WA, USA).

#### 2.4.1. Consistency Test

The consistency of the slurry was measured using a mortar consistency tester (Shanghai Leiyun Test Instrument Manufacturing Co., Ltd., Shanghai, China). Prior to testing, a small amount of lubricating oil was applied to the sliding rod, and both the container and the surface of the test cone were thoroughly moistened. The prepared slurry was then poured into the container in a single operation until the surface was approximately 10 mm below the rim. The slurry was compacted and leveled by gentle insertion, shaking, or tapping, and the container was subsequently placed on the base. The brake screw was released, and after 10 s it was immediately tightened again. The reading at this moment was recorded as the consistency value.

#### 2.4.2. Setting Time Test

The setting time of the slurry was determined using the penetration resistance method. The slurry was poured into the test container until the surface was approximately 10 mm below the rim, and the container was then placed on the pressure gauge base. The test needle was pressed into the slurry to a depth of 25 mm within 10 s, and the corresponding $N_{P}$ was recorded. The penetration resistance value was calculated according to Equation (1), and the relationship between penetration resistance and time was subsequently plotted. The time $t_{s}$ (min) corresponding to a penetration resistance of 0.5 MPa was obtained graphically and defined as the setting time of the slurry.

(1) $$f_{P}=\frac{N_{P}}{A_{P}}$$

where $f_{P}$ is the penetration resistance value (MPa), $N_{P}$ is the static pressure (N) at penetration depth up to 25 mm, and $A_{P}$ is the cross-sectional area (30 mm^2^) of the penetration test needle.

#### 2.4.3. Bleeding Rate Test

A slurry volume of 245 ± 5 mL was poured into a 250 mL graduated cylinder, and the initial scale $a_{0}$ was recorded after the sample had stood for 1 min. After 3 h of standing, the readings of the bleeding surface scale $a_{1}$ and the slurry surface scale $a_{2}$ were taken. The bleeding rate was calculated according to Equation (2) and was required to be less than 5%.

(2) $$BR_{3h}=\frac{a_{1}-a_{2}}{a_{0}}\times 100$$

where $BR_{3h}$ is the 3 h bleeding rate (%), $a_{0}$ is the initial grouting material slurry surface scale value (mL), $a_{1}$ is the bleeding surface scale value (mL) after 3 h, and $a_{2}$ is the slurry surface scale value (mL) after 3 h.

#### 2.4.4. Early-Age Deformation Test

The early-age deformation of the grout was evaluated using the volumetric method, which is defined as the ratio of the difference between the actual volume of grout specimens at various curing ages and the standard mold volume to the standard mold volume. The side lengths of the cubic specimens were measured with a vernier caliper. The early-age deformation coefficient was calculated according to Equation (3).

(3) $$\varepsilon_{t}=\left|\frac{L_{1}L_{2}L_{3}-70.7^{3}}{70.7^{3}}\right|\times 100$$

where $\varepsilon_{t}$ is the early-age deformation coefficient (%) at t days, accurate to 0.01; $L_{1}$, $L_{2}$, $L_{3}$ are the side lengths of the test block at t days.

#### 2.4.5. Compressive Strength Test

The compressive strength test was conducted using a microcomputer-controlled electro-hydraulic testing machine (Shanghai Shenli Testing Machine Co., Ltd., Shanghai, China) at a loading rate of 0.1 kN/s. The compressive strength was calculated according to Equation (4).

(4) $$f_{cu}=\frac{P}{A}$$

where $f_{cu}$ is the unconfined compressive strength of the specimen (MPa), accurate to 0.01; P is failure load (N); and A is the cross-sectional area of the specimen (mm^2^).

#### 2.4.6. X-Ray Diffraction (XRD)

After curing for 28 days, the specimens were split, and representative samples were collected from multiple locations within the central region and thoroughly mixed. The samples were immersed in anhydrous isopropanol to terminate hydration, followed by drying and grinding, and were subsequently stored in sealed containers. The phase composition of the samples was determined by X-ray diffraction (XRD). XRD analysis of the powdered samples was conducted using a Rigaku SmartLab SE diffractometer (Rigaku Corporation, Tokyo, Japan). The measurements were performed in continuous scanning mode over a 2θ range of 5°–90°, with a step size of 0.01° and a scan rate of 10°/min. The instrument was configured in Bragg–Brentano (BB) geometry.

#### 2.4.7. Scanning Electron Microscope (SEM)

After curing for 28 days, the specimens were split, and representative fragments were collected from the central region. The samples were immersed in anhydrous isopropanol to terminate hydration, dried, and subsequently stored in sealed containers. Freshly fractured surfaces were selected for microstructural observation. Before SEM examination, the samples were sputter-coated with gold using an ion sputter coater (Zhengzhou Ketan Instrument Equipment Co., Ltd., Zhengzhou, China), forming an approximately 15 nm thick conductive gold layer on the specimen surfaces. The gold coating was applied to improve surface conductivity and minimize charging artifacts during electron-beam irradiation. SEM observations were performed using a ZEISS Sigma 300 scanning electron microscope (Carl Zeiss, Oberkochen, Germany) in variable-pressure mode with a variable-pressure secondary electron (VPSE) detector at an accelerating voltage of 10 kV. Representative microstructural images were acquired at different observation scales, with scale bars of 2 μm and 4 μm, to characterize the morphology of the hydration products.

## 3. Experiment Results and Analysis

### 3.1. Workability

**Figure 5:**
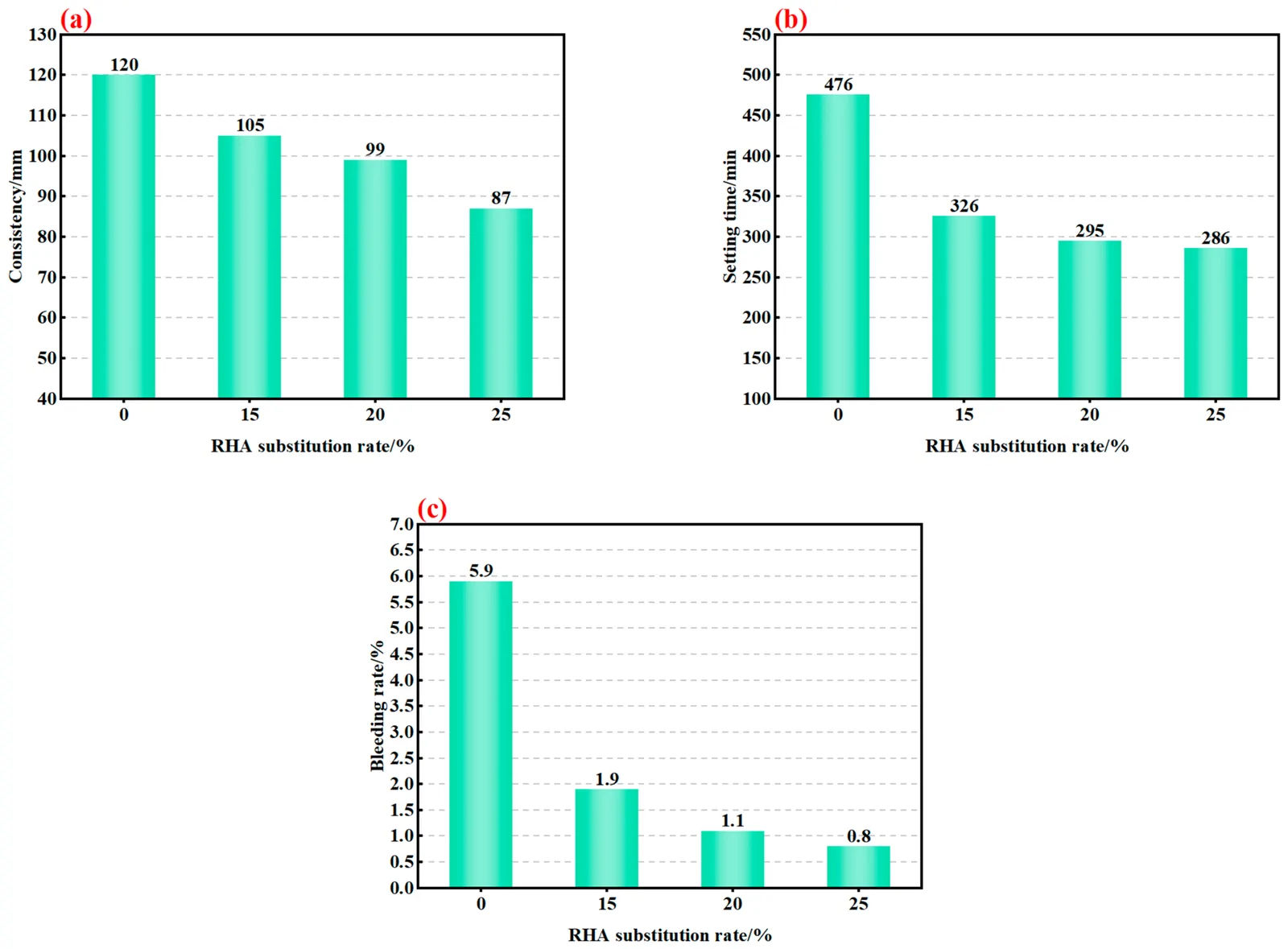
Effect of RHA substitution rate on (**a**) consistency, (**b**) setting time, and (**c**) bleeding rate of grouting materials.

**Figure 6:**
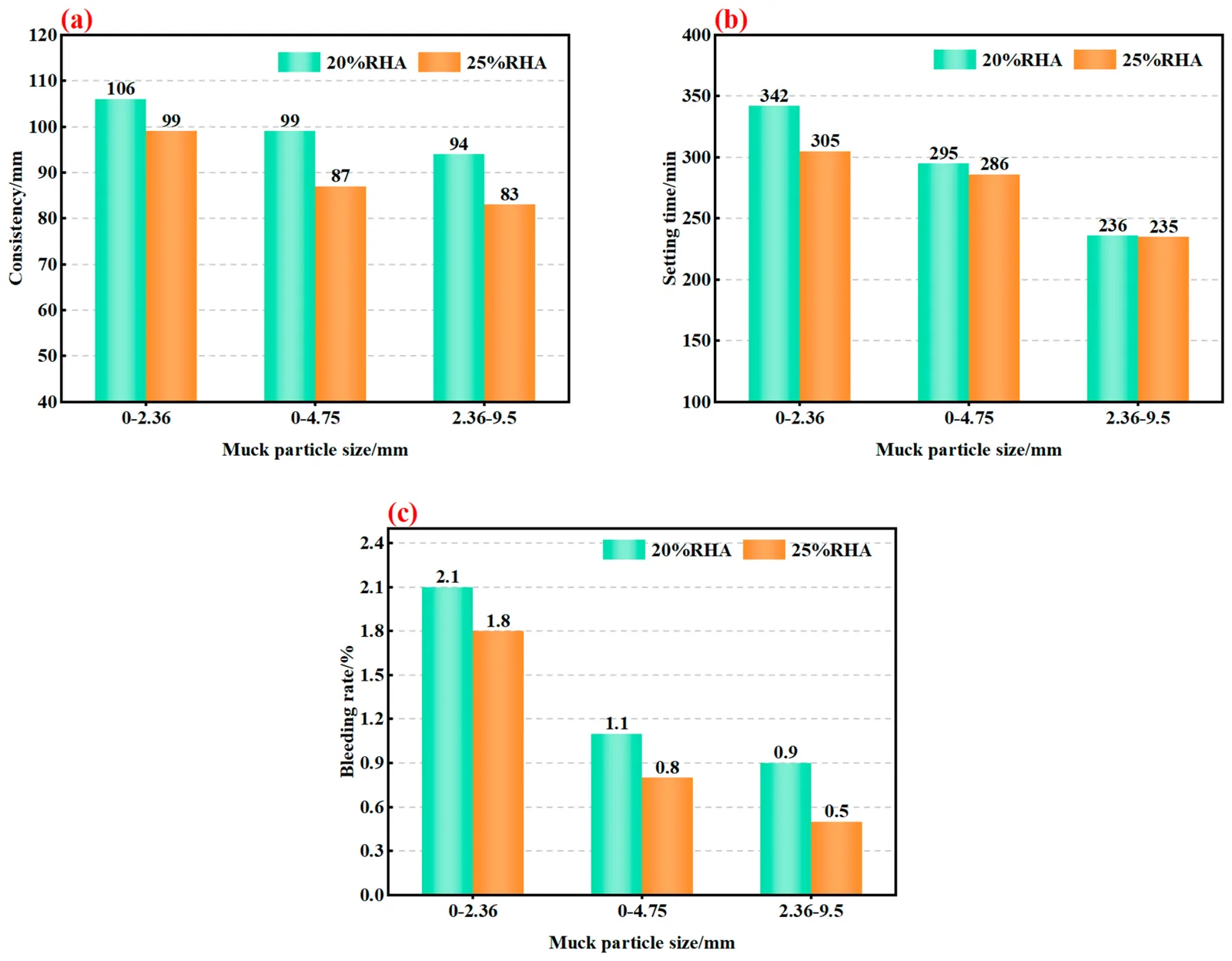
Effect of muck particle size on (**a**) consistency, (**b**) setting time, and (**c**) bleeding rate of grouting materials.

**Figure 7:**
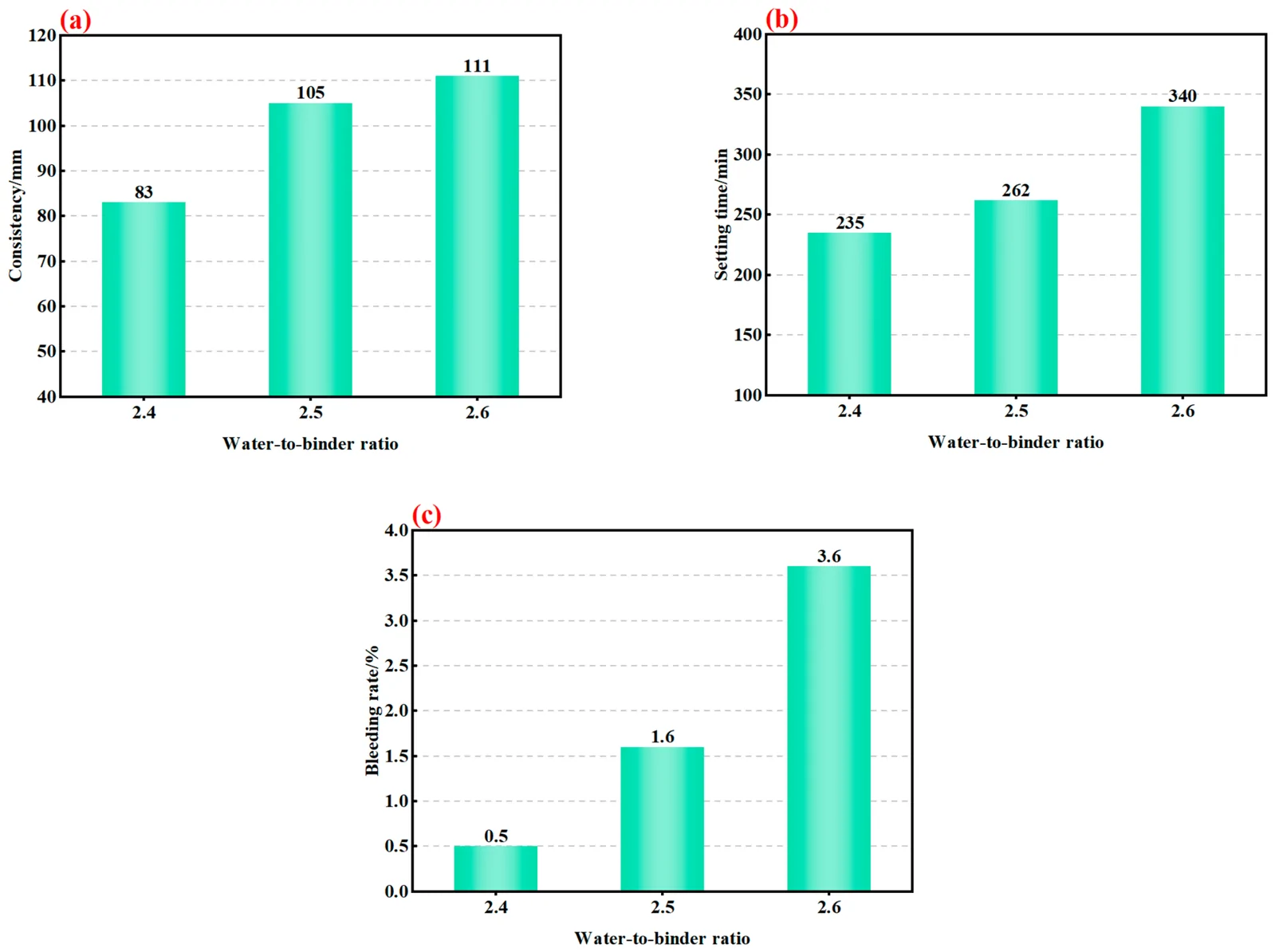
Effect of w/b ratio on (**a**) consistency, (**b**) setting time, and (**c**) bleeding rate of grouting materials.

**Figure 8:**
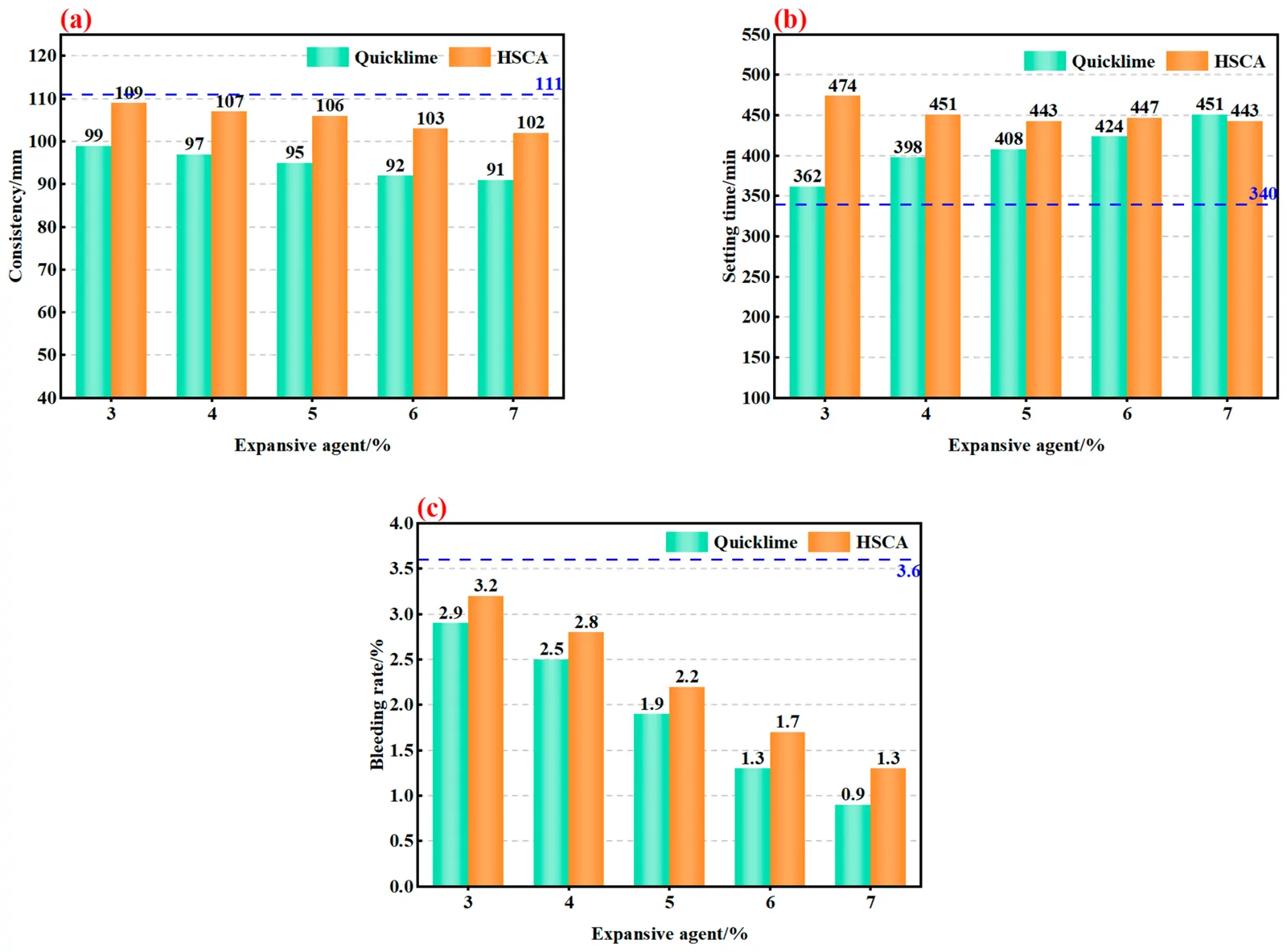
Effect of expansive agents on (**a**) consistency, (**b**) setting time, and (**c**) bleeding rate of grouting materials.

The consistency, setting time, and bleeding rate of the synchronous grouting materials are shown in Figure 5, Figure 6, Figure 7 and Figure 8. The incorporation of RHA led to reductions in the consistency, setting time, and bleeding rate of the grouting materials. At a 25% RHA substitution rate, the consistency of the synchronous grouting material decreased by 27.5%, the setting time was shortened by 39.9%, and the bleeding rate was reduced by 86.4%. In contrast, the addition of the expansive agent caused a slight decrease in consistency, an increase in setting time, and a reduction in bleeding rate.

As the substitution rate of RHA increased, the slurry consistency decreased linearly, as shown in Figure 5a. The water absorption of RHA reduces the free water in the system, leading to a decrease in slurry fluidity and, consequently, a reduction in consistency. Compared with the R0W2.4M group (without RHA), the consistencies of the R15W2.4M, R20W2.4M, and R25W2.4M groups decreased by 12.5%, 17.5%, and 27.5%, respectively. The incorporation of RHA also accelerated the setting of the slurry, as illustrated in Figure 5b. The setting time decreased with increasing RHA content. This effect is attributed to the promotion of C_3_S hydration by RHA [33], which enhances the formation rate of C-S-H and Ca(OH)_2_ [34], thereby shortening the setting time. Compared with the R0W2.4M group, the setting times of the R15W2.4M, R20W2.4M, and R25W2.4M groups were reduced by 31.5%, 38.0%, and 39.9%, respectively. Furthermore, RHA substantially improved the bleeding performance of the grouting material, as shown in Figure 5c. Compared with the R0W2.4M mixture, the bleeding rates of R15W2.4M, R20W2.4M, and R25W2.4M decreased by 67.8%, 81.4%, and 86.4%, respectively, with the bleeding rate of R25W2.4M reaching 0.8%.

As the muck particle size increased, the consistency, setting time, and bleeding rate of the slurry gradually decreased, as shown in Figure 6. At a 20% RHA substitution rate, the consistency values of the slurries with medium and large muck particle sizes were 93.8% and 88.7% of that with small particles, respectively; the setting times were 86.3% and 69.0% of that with small particles, respectively; and the bleeding rates were 52.3% and 42.9% of that with small particles, respectively. At a 25% RHA substitution rate, the consistency values of the slurries with medium and large particle sizes decreased to 87.9% and 83.8% of that with small particles, the setting times were 93.8% and 77.0% of that with small particles, and the bleeding rates were 44.4% and 27.8% of that with small particles, respectively. These trends can be attributed to the superior water retention of larger particles, which consume more water and reduce the amount of free water in the system. This leads to a decrease in slurry fluidity and an acceleration of setting. Furthermore, for small particle size muck, the setting time of the slurry containing 25% RHA was 89.2% of that with 20% RHA. In contrast, for medium and large particle sizes, the setting times were nearly identical. This behavior occurs because, as the muck particle size increases, its water absorption gradually exceeds that of RHA; the combined water absorption rapidly reduces free water in the slurry, thereby diminishing the effect of RHA on the setting time.

Figure 7 illustrates the effects of the water-to-binder ratio on the consistency, setting time, and bleeding rate of the slurry. As the w/b ratio increased, the consistency value increased markedly. Specifically, the consistency of the R25W2.6L mixture increased from 83 mm to 111 mm compared with that of R25W2.4L, corresponding to an increase of 33.7%. This behavior was attributed to the greater amount of free water in the slurry, which reduced interparticle friction and cohesion and thereby improved slurry flowability. The setting time also increased with increasing w/b ratio; the setting time of R25W2.6L was 44.7% longer than that of R25W2.4L. At a lower w/b ratio, the available water was consumed more rapidly during cement hydration, resulting in a shorter setting time, whereas the additional free water at a higher w/b ratio delayed setting. In addition, the bleeding rate increased continuously with the w/b ratio. The bleeding rates of R25W2.5L and R25W2.6L were 2.2 and 6.2 times that of R25W2.4L, respectively. This increase was mainly caused by the greater free-water content and the reduced stability of the solid–liquid suspension. Nevertheless, the bleeding rates of all mixtures remained within the allowable range for construction applications.

As the dosage of the expansive agent increased, the slurry consistency gradually decreased and remained lower than that of the slurry without the expansive agent, as shown in Figure 8a. The strong water absorption of CaO (theoretical water absorption rate of 32.14%) reduces the free water content in the system, resulting in a decrease in slurry consistency. After the addition of quicklime, the consistency decreased by 10.8%, 12.6%, 14.4%, 17.1%, and 18.0% compared to the baseline group (R25W2.6L). The main source of expansion in HSCA is overburnt lime, which reacts more slowly with water; thus, the consistency of HSCA-slurry was slightly higher than that of the quicklime-slurry. After adding HSCA, the consistency decreased by 1.8%, 3.6%, 4.5%, 7.2%, and 8.1% relative to the baseline group. The addition of the expansive agent also affected the setting time, as shown in Figure 8b. For quicklime-slurry, the setting time gradually increased with increasing dosage, showing increments of 6.5%, 17.1%, 20.0%, 24.7%, and 32.6% compared to the baseline. In contrast, the setting time of HSCA-slurry was substantially higher than that of the baseline group but was relatively insensitive to the HSCA dosage. Similarly, the bleeding rate decreased with the addition of the expansive agent, as shown in Figure 8c. For quicklime-slurry, the bleeding rate decreased by 19.4%, 30.6%, 47.2%, 63.9%, and 75.0%, while for HSCA-slurry, the reductions were 11.1%, 22.2%, 38.9%, 52.8%, and 63.9% relative to the baseline. This behavior is closely related to the water absorption capacity of the expansive agent: quicklime has a higher water absorption than HSCA, consuming more free water during hydration and thereby reducing the amount of water exuded.

### 3.2. Early-Age Deformation

During the curing process, grouting materials may undergo volume shrinkage due to water loss, which can result in the formation of cavities behind segments in practical engineering applications. The incorporation of RHA substantially reduced the early-age volume shrinkage ratio of the grouting material. The lowest volume shrinkage ratio was observed at a 25% RHA substitution rate, with a reduction of 78.2%. To mitigate such shrinkage and prevent potential engineering issues, various types of expansive agents can be added to the slurry. These agents not only compensate for volume shrinkage but also induce slight expansion, leading to a more compact and dense filling.

**Figure 9:**
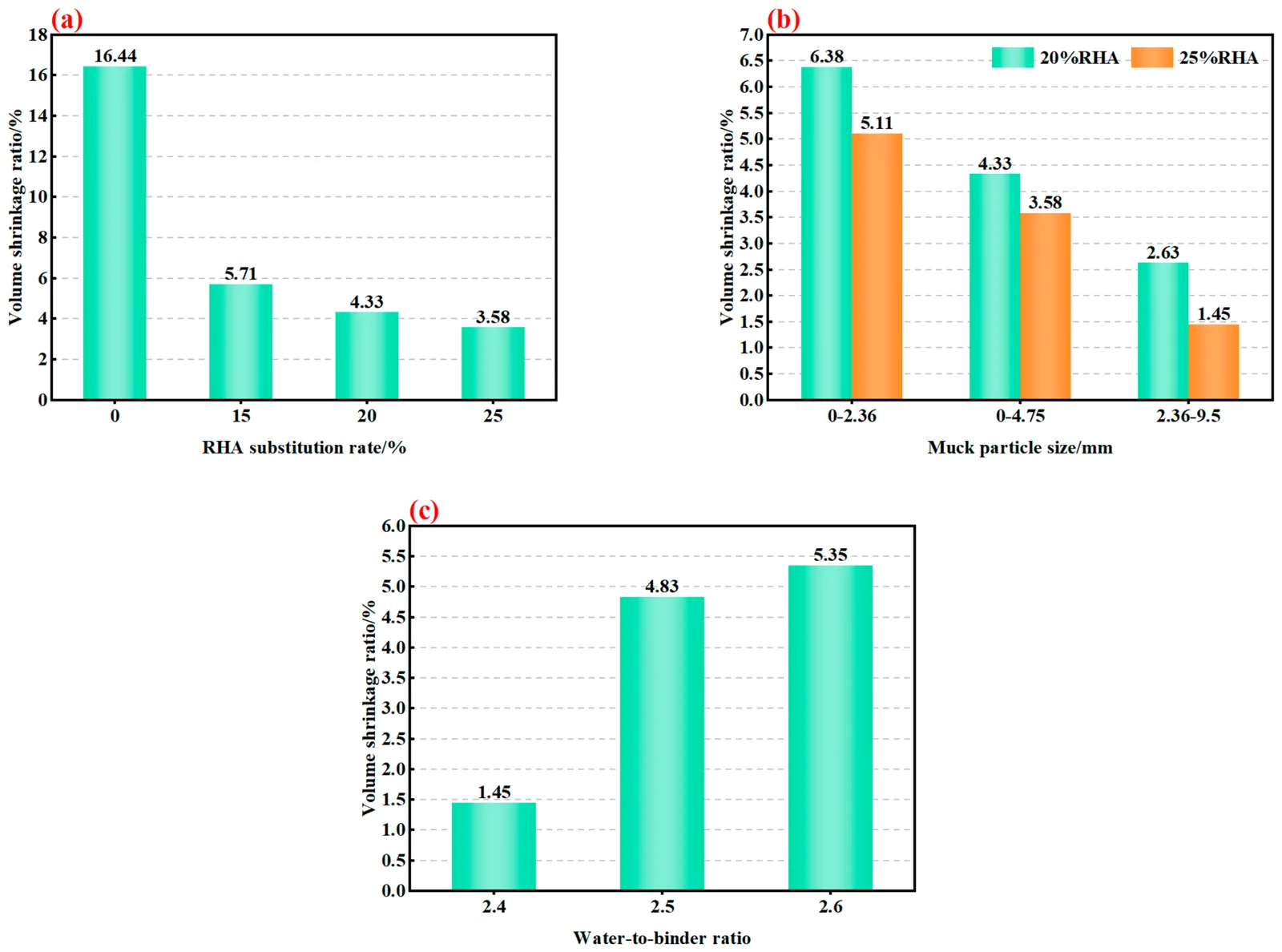
Effect on volume shrinkage ratio of grouting materials: (**a**) RHA substitution rate; (**b**) muck particle size; (**c**) w/b ratio.

Figure 9 presents the effects of the RHA substitution rate, shield muck particle size, and water-to-binder ratio on the volume shrinkage of the grouting materials. As shown in Figure 9a, increasing the RHA substitution rate markedly reduced volume shrinkage, although the magnitude of improvement gradually diminished at higher substitution rates. Compared with the R0W2.4M reference mixture, the volume shrinkage ratios of R15W2.4M, R20W2.4M, and R25W2.4M decreased by 65.3%, 73.7%, and 78.2%, respectively. This reduction can be attributed to the water absorption and retention capacity of RHA, which limits moisture loss from the slurry. Figure 9b shows that volume shrinkage also decreased with increasing shield muck particle size. At an RHA substitution rate of 20%, the shrinkage ratios of the mixtures containing medium and large particles of muck were 67.9% and 41.2%, respectively, of that containing small particles of muck; at an RHA substitution rate of 25%, the corresponding values were 70.1% and 28.4%. The greater shrinkage associated with smaller particles was attributed to their higher capillary porosity, which generated greater capillary stress during moisture loss. In contrast, increasing the w/b ratio substantially increased volume shrinkage, as shown in Figure 9c. The shrinkage ratios of R25W2.5L and R25W2.6L were 3.33 and 3.69 times that of R25W2.4L, respectively. A higher w/b ratio increased the amount of free water and capillary porosity within the hardened matrix, thereby intensifying capillary stress and volume shrinkage during moisture loss [35,36].

**Figure 10:**
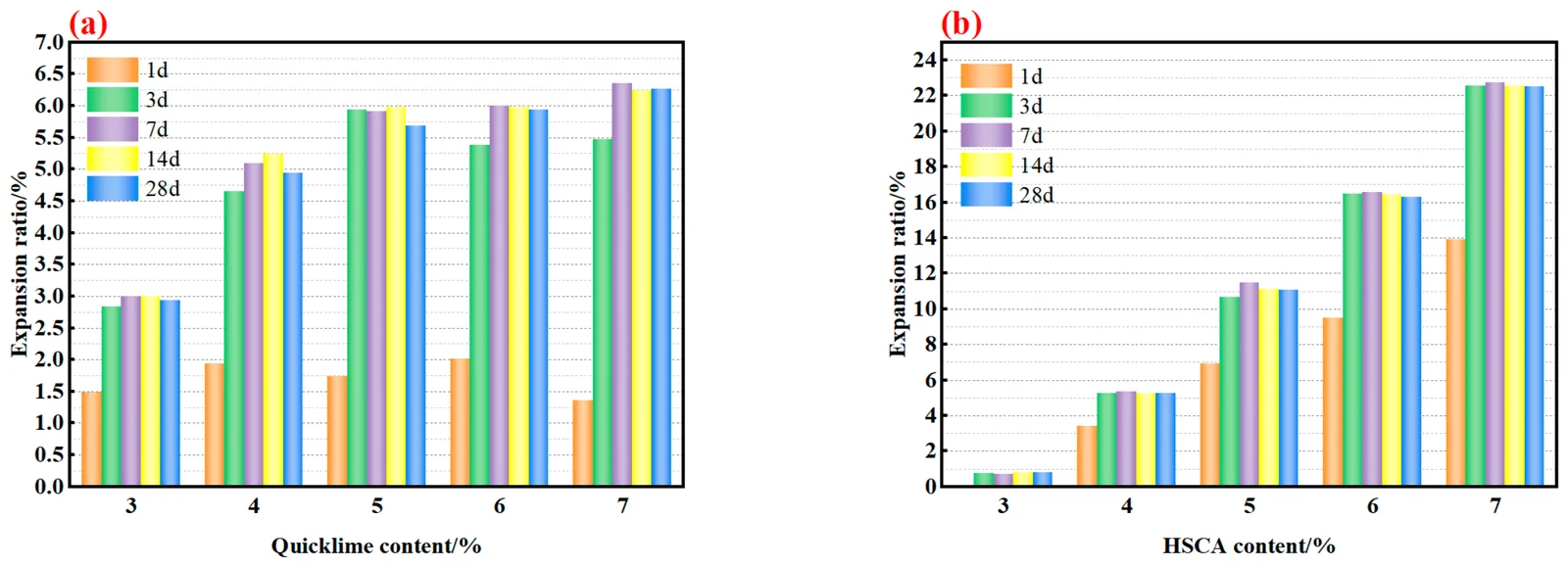
Effect of expansive agent dosage on expansion ratio of grouting materials: (**a**) quicklime; (**b**) HSCA.

The expansion ratios at 1, 3, 7, 14, and 28 days after the addition of the expansive agents are shown in Figure 10a,b. The expansion of the grouting materials increased with higher dosages of the expansive agent. Overall, slurries mixed with HSCA exhibited higher expansion ratios than those mixed with quicklime. At low dosages of the expansive agent (≤3%), the expansion ratio of HSCA-slurry was very low, even lower than that of quicklime-slurry. However, when the dosage exceeded 4%, the expansion capacity of HSCA increased markedly, reaching rates 2 to 4 times higher than those of quicklime. Although both expansive agents derive their expansion primarily from CaO, and quicklime contains a higher CaO content, the test results indicate that the expansion capacity of quicklime is substantially lower than that of HSCA.

### 3.3. Compressive Strength

The incorporation of RHA had little effect on the compressive strength of the grouting materials. At a 25% substitution rate, the 3-day and 28-day compressive strengths remained essentially unchanged compared with the control sample. In contrast, the addition of expansive agents led to an improvement in the compressive strength at both 3-day and 28-day ages, although the degree of enhancement varied slightly. Moreover, at the same dosage, different types of expansive agents produced approximately the same level of strength enhancement.

**Figure 11:**
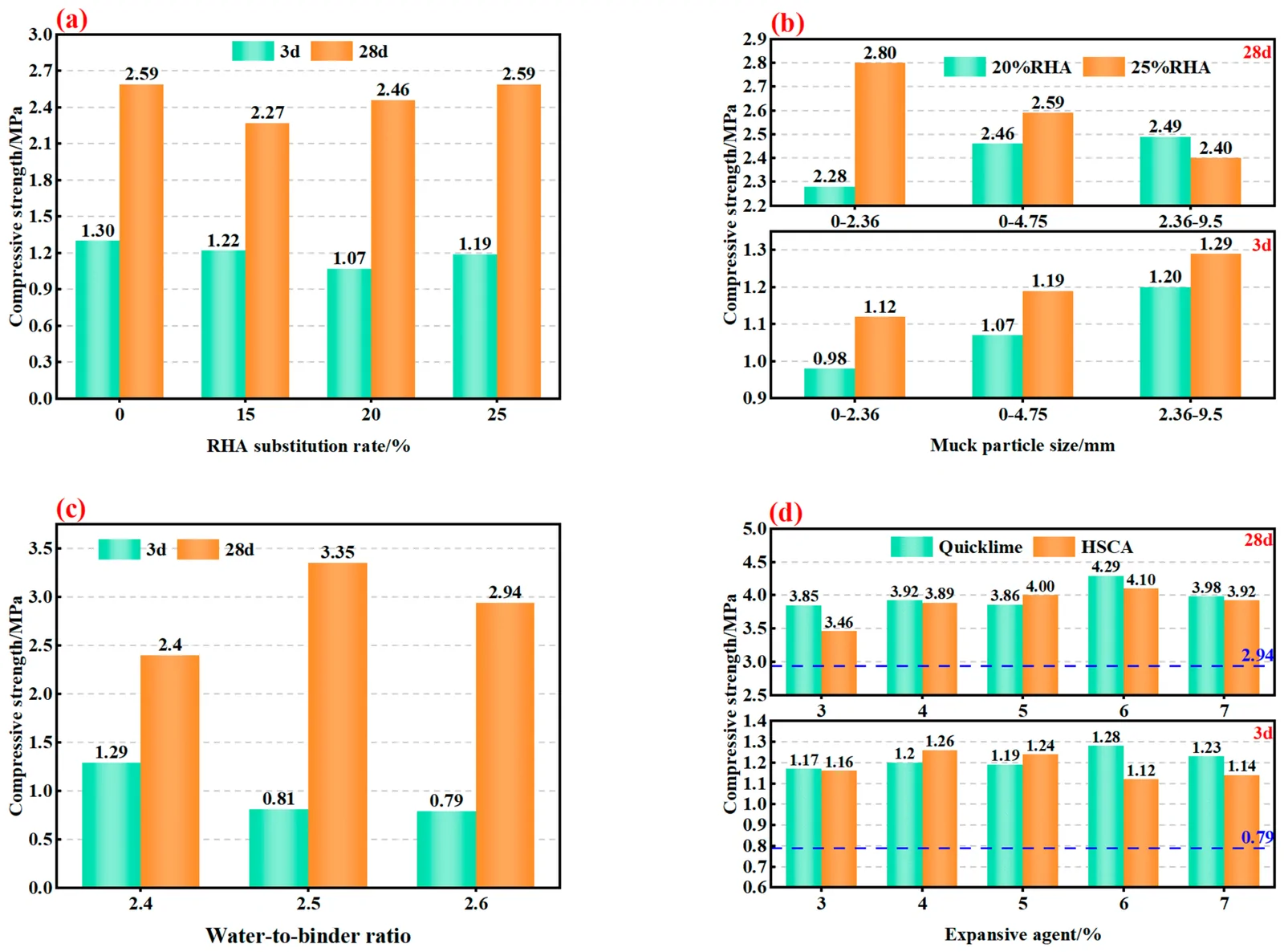
Effect on compressive strength of grouting materials: (**a**) RHA substitution rate; (**b**) muck particle size; (**c**) w/b ratio; (**d**) expansive agent.

Figure 11 illustrates the effects of RHA substitution rate, muck particle size, w/b ratio and expansive agents on the compressive strength of the slurry. As shown in Figure 11a, the slurry strength initially decreases and then increases with increasing RHA substitution rate at both 3-day and 28-day ages. At substitution rates of 20% and 15%, the 3-day and 28-day strengths reached minimum values, decreasing by 17.7% and 12.4% compared with the R0W2.4M group, respectively. However, at a 25% substitution rate, the 3-day and 28-day strengths increased by 11.2% and 14.1%, respectively, relative to the corresponding lower substitution groups. This trend is attributed to the water absorption and lower density of RHA, which initially increase porosity and reduce compactness, lowering strength. At higher substitution rates, the enhanced pozzolanic reaction between RHA SiO_2_ and cement hydration product Ca(OH)_2_ promotes further hydration, compensating for the initial strength loss [37,38].

Figure 11b shows the influence of muck particle size on compressive strength. Early-age strength (3-day) increases with larger particle sizes. For a 20% RHA substitution rate, the 28-day strength continues to rise with increasing particle size, reflecting the beneficial effect of improved particle gradation on packing density and strength. In contrast, at a 25% RHA substitution rate, the 28-day strength decreases with increasing particle size, falling below that of the 20% RHA slurry at larger sizes. This is because larger muck particles retain water more effectively, limiting free water availability for RHA hydration and reducing cement hydration. Smaller particles, which retain less water, allow RHA to utilize the available water, enhancing later-age strength. At 28 days, the small particle size slurry with 25% RHA achieves 1.17 times the strength of the large particle size slurry and 1.23 times that of the 20% RHA slurry.

Figure 11c shows the effect of the w/b ratio on the compressive strength of the grouting materials. As the w/b ratio increased, the 3-day compressive strength decreased from 1.29 MPa to 0.79 MPa, corresponding to a reduction of 38.8%. In contrast, the 28-day compressive strength initially increased and then decreased, reaching a maximum value of 3.35 MPa at a w/b ratio of 2.5. A moderate increase in the w/b ratio provided sufficient water for the continued hydration and pozzolanic reaction of RHA, thereby promoting later-age strength development. However, an excessively high w/b ratio introduced excess free water and increased capillary porosity after hardening, resulting in a reduction in compressive strength.

The effect of expansive agents is presented in Figure 11d. Both 3-day and 28-day strengths are substantially enhanced by the addition of expansive agents compared with the baseline group (R25W2.6L). At 28 days, the compressive strength of the slurry mixed with quicklime increased by 31.0%, 33.3%, 31.3%, 45.9%, and 35.4%, respectively, while that of the slurry mixed with HSCA increased by 17.7%, 32.3%, 36.1%, 39.5%, and 33.3%, respectively. The strength enhancement is attributed to the crystallization-induced expansion of the slurry being constrained by the mold, which generates internal pre-stress, resulting in a denser microstructure and higher compressive strength.

### 3.4. Microstructure Characteristics

Four representative mixtures were selected for the XRD and SEM analyses based on their compositions, roles in the experimental program, and macroscopic performance. R0W2.4M was selected as the unmodified reference mixture to provide a baseline for characterizing the hydration products and microstructure of the shield muck-based grouting material. R25W2.6L was selected as the representative modified base mixture and served as the reference mixture for the subsequent expansive-agent tests. R25W2.6LC7 and R25W2.6LH7 were selected as representative mixtures containing 7% quicklime and 7% HSCA, respectively, to characterize the microstructural effects associated with the two types of expansive agents. Comparison of these four mixtures enabled the microstructural changes associated with RHA modification and expansive agent incorporation to be examined and provided a basis for interpreting the observed differences in volumetric deformation and mechanical performance.

#### 3.4.1. XRD Analysis

**Figure 12:**
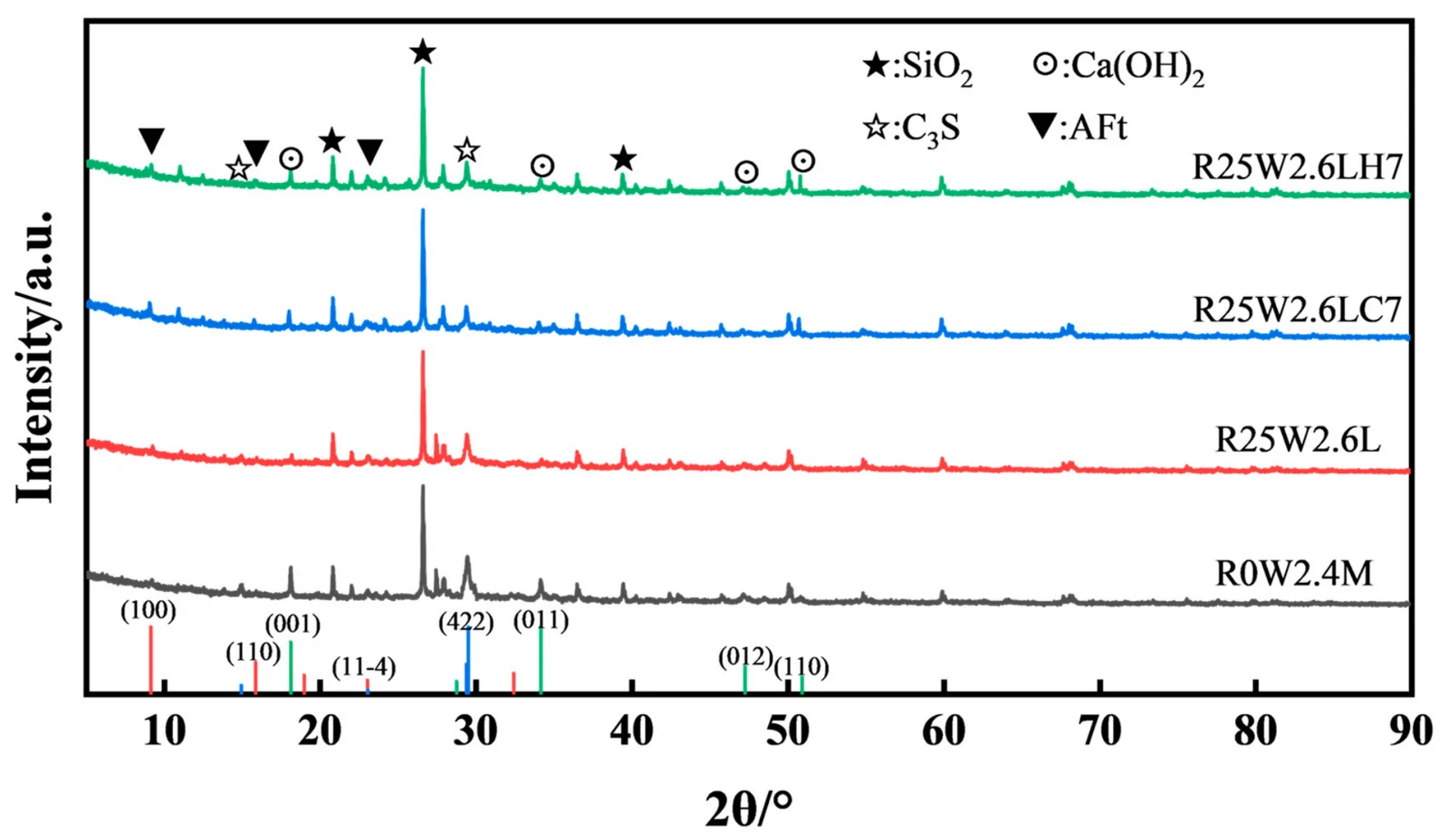
XRD patterns of R0W2.4M, R25W2.6L, R25W2.6LC7, and R25W2.6LH7 groups.

The XRD patterns of samples from groups R0W2.4M, R25W2.6L, R25W2.6LC7, and R25W2.6LH7 are presented in Figure 12. In the R0W2.4M samples, the diffraction peak intensities of C_3_S (tricalcium silicate) at the (310) and (422) planes are markedly lower than those in the R25W2.6L samples, while the diffraction peaks of Ca(OH)_2_ at the (001), (011), and (110) planes are substantially higher. This suggests that RHA promotes the hydration of C_3_S by consuming Ca(OH)_2_ generated during cement hydration, leading to the formation of more flake-like C-S-H, which in turn enhances the mechanical properties of the grouting material. Furthermore, the diffraction peaks of AFt (ettringite) at the (100), (110), and (11-4) planes in the R25W2.6LC7 and R25W2.6LH7 samples are slightly more intense than those in the R25W2.6L samples. Considering the notable expansion characteristics of AFt, its formation can be regarded as a contributing factor to the expansion behavior of the grouting material.

#### 3.4.2. SEM Analysis

**Figure 13:**
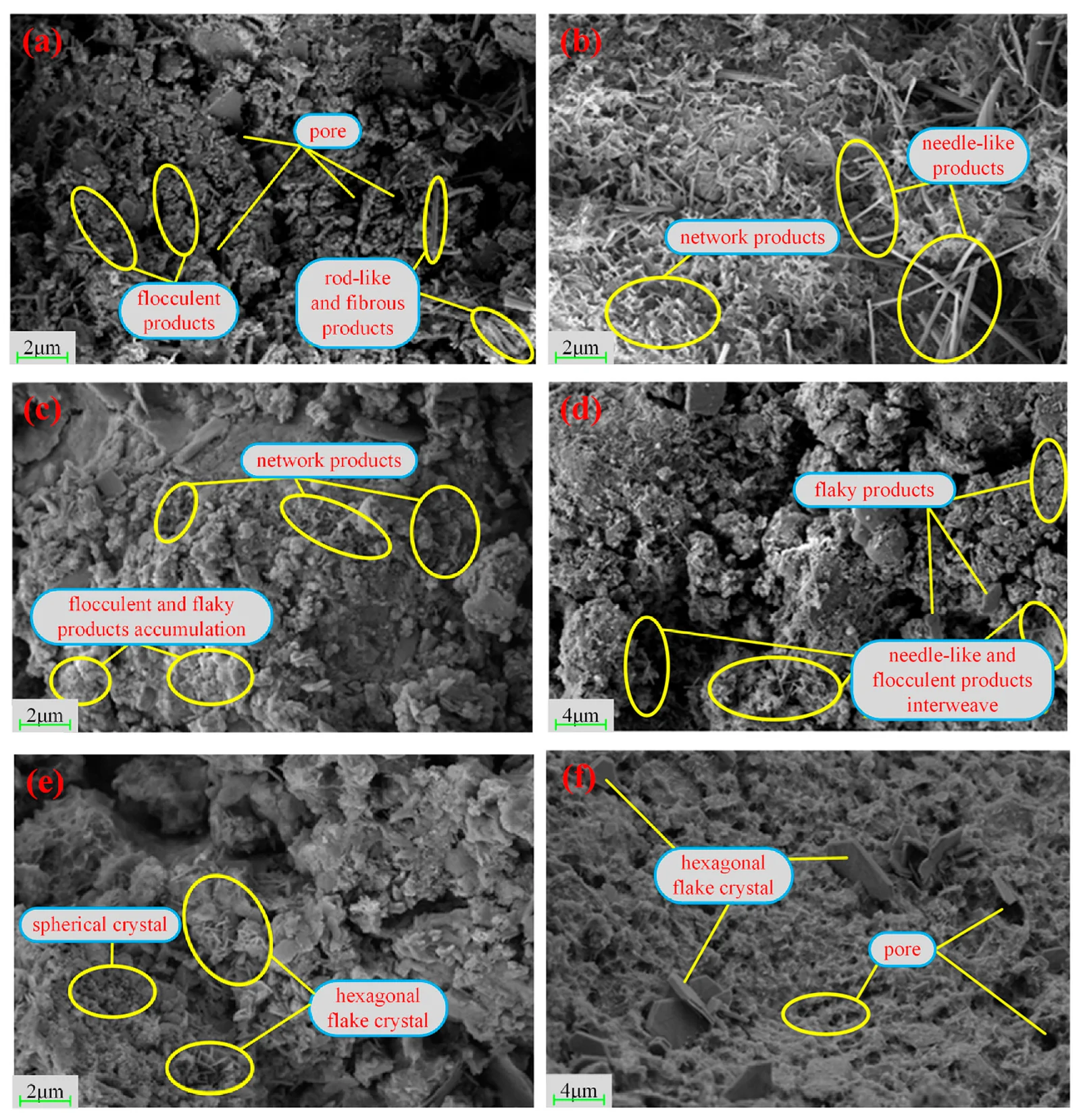
SEM images of (**a**,**b**) R0W2.4M, (**c**,**d**) R25W2.6L, (**e**) R25W2.6LC7 and (**f**) R25W2.6LH7 groups.

Figure 13a,b show the microstructure of the R0W2.4M sample (without RHA). The hydration products are diverse, primarily consisting of flocculent and reticular C-S-H gel (calcium silicate hydrate) and needle-like or rod-like AFt (ettringite). The flocculent gel wraps around soil particles and fills the interparticle pores, while the reticular gel connects the soil particles and fillers into a network-like structure. The needle-like and fibrous products are irregularly interspersed among the soil particles and gel, reinforcing structural connections. Overall, the structure exhibits numerous pores and loose connections, resulting in relatively low compressive strength after curing.

Figure 13c,d present the microstructure of the R25W2.6L sample (with 25% RHA substitution). Compared with the R0W2.4M sample, the incorporation of RHA promotes the formation of additional flocculent and lamellar gels, which adhere to the reticular gels and accumulate, leaving only limited reticular gel visible. The newly formed gels fill pores and microcracks between soil particles, forming aggregates that enhance structural compactness. Numerous needle-like AFt and flocculent gels intertwine, reducing the observable amount of needle-like products. These observations indicate that RHA substantially promotes cement hydration, increases the quantity of hydration products, densifies the slurry microstructure, reduces porosity, and improves post-curing strength.

Figure 13e,f show the microstructure of the R25W2.6LC7 (7% quicklime) and R25W2.6LH7 (7% HSCA) samples under free expansion conditions, respectively. Both slurries exhibit abundant Ca(OH)_2_ crystals, consistent with their XRD results. In Figure 13e, numerous hexagonal plate-like and spherical Ca(OH)_2_ crystals are clustered together. The grouting material undergoes two stages of expansion during hydration: first, at the initial cement hydration stage, the expansive agent reacts with water to form gel-like Ca(OH)_2_; second, the early-formed Ca(OH)_2_ recrystallizes into hexagonal crystals, further increasing the volume. In Figure 13f, the grouting material contains dispersed Ca(OH)_2_ crystals, which are fewer than in the R25W2.6LC7 sample. The R25W2.6LH7 system shows a uniform gel distribution with numerous fine pores, which drives internal expansion and explains why HSCA exhibits a higher expansion ratio than quicklime.

## 4. Discussion

**Figure 14:**
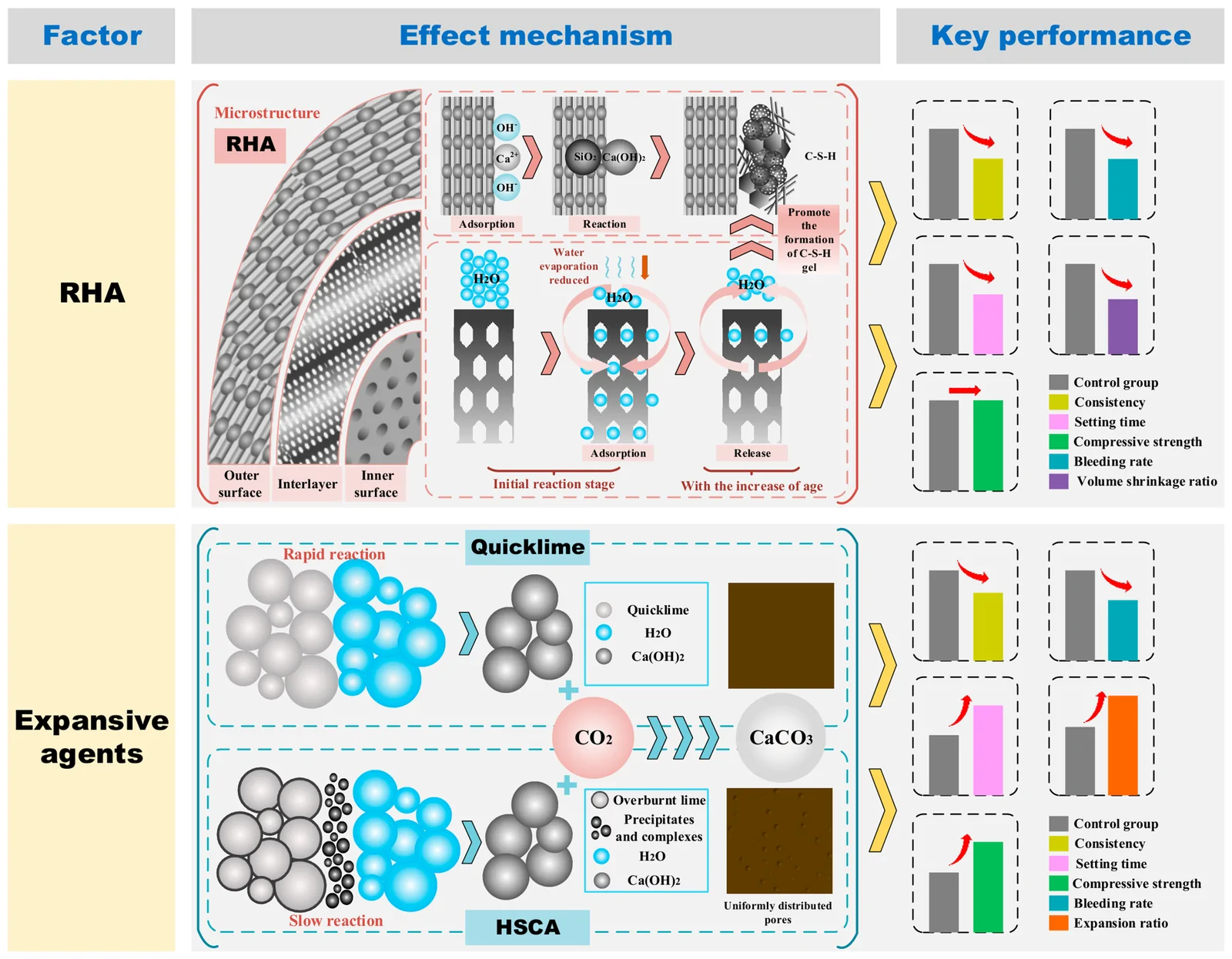
Effect of different factors on the grouting materials.

As shown in Figure 14, RHA exhibits a porous honeycomb-like structure composed of interconnected platelets and fine particles, with numerous internal voids [39]. This morphology provides RHA with a high water-absorption capacity, thereby reducing the free-water content as the RHA substitution rate increases [40]. The reduced free-water content increases interparticle resistance and consequently decreases the consistency value of the slurry. A similar reduction in workability has been reported for conventional RHA-containing concrete, in which increasing the RHA substitution rate from 0% to 20% markedly reduced the slump because of the high specific surface area and water demand of RHA [41]. The present results demonstrate that this behavior also occurs in shield muck-based grouting materials.

Although RHA has been reported to prolong the setting of conventional Portland cement systems [42], the setting time decreased in the present study. This difference may be related to the presence of fine soil particles in the shield muck. The absorption of free water by RHA promoted the dehydration and consolidation of these particles, which may have dominated the setting behavior of the investigated slurry. Therefore, the effects of RHA on setting time depend not only on cement hydration but also on water redistribution and soil-particle consolidation within the shield muck-based system.

The porous structure of RHA also contributed to water retention and reduced moisture migration from the slurry. Consequently, bleeding and early-age volume shrinkage decreased as the RHA substitution rate increased. Compared with the R0W2.4M reference mixture, the volume shrinkage of R15W2.4M, R20W2.4M, and R25W2.4M decreased by 65.3%, 73.7%, and 78.2%, respectively. Previous research on RHA-containing cement pastes reported reductions in autogenous shrinkage of approximately 23.8–41.6%, depending on the particle size of the RHA [20]. Although the reduction obtained in the present study was greater, the values should not be interpreted as a direct indication of superior performance because the two studies involved different material systems, shrinkage indices, mixture proportions, and curing conditions. The relatively large reduction observed here may be attributed to the combined effects of water retention by RHA, the internal restraint provided by the shield muck skeleton, and the relatively high initial shrinkage of the unmodified reference mixture.

During hydration, Ca^2+^ and OH^−^ ions are adsorbed on the porous surface of RHA particles. The reactive SiO_2_ in RHA subsequently reacts with Ca(OH)_2_ to form additional C–S–H gel [19,43,44]. Meanwhile, water absorbed by RHA at the early stage can be gradually released as the internal relative humidity decreases [21,34]. This internal curing effect helps maintain hydration, partially compensates for moisture loss, and promotes later-age C–S–H formation. The newly formed hydration products fill the pores and improve matrix continuity [21,45], consistent with the XRD and SEM observations.

However, the compressive strength did not increase monotonically with the RHA substitution rate. The 28-day compressive strength decreased by 12.4% at an RHA substitution rate of 15% and subsequently recovered as the substitution rate increased to 25%. Previous research has reported that ultrafine RHA at a substitution rate of 20% increased the 28-day compressive strength by approximately 15%, whereas a substitution rate of 15% produced an increase of only approximately 4% [41]. The less pronounced strength enhancement in the present study may be associated with the lower cement content and the incorporation of shield muck, which increased water demand and reduced the proportion of reactive binder. At intermediate RHA substitution levels, the dilution of cement and incomplete pozzolanic reaction may have outweighed the filler and internal curing effects. At the highest investigated substitution rate, the combined contribution of water retention, secondary C–S–H formation, and pore filling promoted the recovery of later-age strength [19,46,47]. These comparisons confirm that the mechanical contribution of RHA depends strongly on its substitution rate, fineness, reactivity, available water, and the composition of the matrix.

The incorporation of quicklime and HSCA further modified the performance of the RHA-containing mixtures. Hydration of the CaO component produced Ca(OH)_2_ and generated crystallization induced expansion, which compensated for volumetric contraction. The formation of solid hydration products also restricted slurry flow, thereby reducing the consistency value. Under the investigated conditions, increasing the expansive agent dosage prolonged the setting time, suggesting that the changes in water consumption and pore solution conditions affected the effective setting process. Subsequent carbonation of Ca(OH)_2_ may also form CaCO_3_ and contribute to matrix densification, although this contribution depends on the curing and exposure conditions [48,49].

The different expansion behaviors of quicklime and HSCA can be attributed primarily to their hydration kinetics. Quicklime reacted rapidly after contact with water, and part of its expansion occurred before the slurry was placed in the test mold. Consequently, only part of its total expansion was recorded during curing. In contrast, the CaO component in HSCA was less reactive at the initial stage because of its overburnt structure and the presence of hydration regulating additives. Its expansion therefore occurred mainly after placement. This delayed expansion behavior is consistent with previous studies on HSCA-based expansive cementitious materials, in which the controlled hydration of overburnt CaO was reported to shift a substantial portion of the expansion to the post-placement stage [13,50]. After hydration, HSCA generated numerous uniformly distributed pores within the slurry matrix, thereby further increasing the overall volumetric expansion ratio [50]. When the dosage exceeded 4%, the expansion ratio produced by HSCA was approximately 2 to 4 times that produced by quicklime under comparable conditions. This delayed expansion is more favorable for compensating for post-placement shrinkage; however, the expansive agent dosage must be controlled to avoid excessive expansion and an undesirable extension of the setting time.

Overall, the results indicate that RHA and expansive agents perform complementary functions in shield muck-based grouting materials. RHA mainly regulates water distribution, reduces bleeding and shrinkage, and supports later-age hydration, whereas quicklime and HSCA provide additional shrinkage compensation through crystallization induced expansion. Nevertheless, mixture selection requires a balance among workability, setting time, bleeding, early-age deformation, and compressive strength. Within the investigated range, the use of 25% RHA combined with large particle size shield muck and a controlled expansive agent dosage provided a practical balance among workability, shrinkage compensation, and mechanical performance. These findings provide a potential approach for reducing shrinkage induced filling defects in shield-tail grouting while increasing the utilization of shield muck and agricultural waste. However, this practical implication should be verified through field-scale grouting tests, long-term deformation monitoring, and durability evaluation under actual tunneling conditions.

## 5. Conclusions

This study investigated the effects of the RHA substitution rate, shield muck particle size, w/b ratio, and expansive agent type and dosage on the workability, early-age deformation, mechanical properties, and microstructure of shield muck-based grouting materials. Within the investigated parameter range, the main conclusions are as follows:

(1) The incorporation of RHA altered the workability of the grouting materials because of its high water-absorption capacity. The consistency value and setting time decreased by 12.5–27.5% and 31.5–39.9%, respectively. RHA also improved early-age volumetric stability. Compared with the R0W2.4M reference mixture, the volume shrinkage of the modified mixture was reduced by up to 78.2%.
(2) The compressive strength did not increase continuously with the RHA substitution rate. It initially decreased at intermediate substitution levels and subsequently recovered or increased slightly at the highest substitution rate investigated. This result indicates that the mechanical performance of RHA-modified grouting materials depends on the balance among the RHA substitution rate, available water, and hydration conditions.
(3) Shield muck particle size substantially affected both the fresh and hardened properties of the grouting materials. Small particle size muck increased capillary porosity, resulting in higher consistency values, longer setting times, and lower early-age strength. In contrast, large particle size muck improved particle packing and was generally more favorable for compressive strength. A relatively high w/b ratio was also beneficial for maintaining sufficient moisture for RHA hydration.
(4) The addition of quicklime or HSCA further improved the volumetric stability and compressive strength of the RHA-modified mixtures. Both expansive agents reduced the consistency value and prolonged the setting time. When the dosage exceeded 4%, HSCA produced an expansion ratio approximately 2 to 4 times that obtained with quicklime under comparable conditions.
(5) Within the investigated range, the 6% quicklime and 6% HSCA mixtures exhibited the highest 28-day compressive strengths, whereas the 7% mixtures produced the greatest expansion. Therefore, the expansive-agent dosage should be selected according to the required balance between shrinkage compensation, strength, setting time, and allowable expansion.

The combined use of RHA, appropriately graded shield muck, and expansive agents provides a feasible approach to improving the volumetric stability and engineering performance of shield muck-based grouting materials while promoting the reuse of shield muck and agricultural waste. However, the liquid limit, plastic limit, and plasticity index of the shield muck were not determined in the present study, which limits a more comprehensive evaluation of the effects of soil plasticity and water sensitivity on the workability and volumetric stability of the grouting materials. Future research should systematically characterize the Atterberg limits of shield muck and clarify their relationships with water demand, bleeding, early-age deformation, and mechanical performance. Further investigations are also needed to evaluate the long-term durability, field-scale performance, and environmental impacts of the proposed grouting materials under practical tunneling conditions. Life-cycle assessment and cost analysis would further clarify their engineering feasibility and sustainability.

## Figures and Tables

**Table 1:** Basic properties of cement.

Cement Grade	Specific Surface Area/(m^2^·kg^−1^)	Setting Time/min		3-Day Strength/MPa	
		Initial Setting Time	Final Setting Time	Flexural Strength	Compressive Strength
P.O. 42.5	343	217	267	6.3	32.5

**Table 2:** Mix proportion design of shield muck-based grouting materials.

Sample No.	RHA Substitution Rate/%	w/b	Particle Size/mm	Quicklime/%	HSCA/%
R0W2.4M	0	2.4	0–4.75	—	—
R15W2.4M	15	2.4	0–4.75		
R20W2.4M	20	2.4	0–4.75		
R25W2.4M	25	2.4	0–4.75		
R20W2.4S	20	2.4	0–2.36		
R20W2.4L	20	2.4	2.36–9.5		
R25W2.4S	25	2.4	0–2.36		
R25W2.4L	25	2.4	2.36–9.5		
R25W2.5L	25	2.5	2.36–9.5		
R25W2.6L	25	2.6	2.36–9.5		
R25W2.6LC3	25	2.6	2.36–9.5	3	—
R25W2.6LC4				4	
R25W2.6LC5				5	
R25W2.6LC6				6	
R25W2.6LC7				7	
R25W2.6LH3				—	3
R25W2.6LH4					4
R25W2.6LH5					5
R25W2.6LH6					6
R25W2.6LH7					7

**Table 3:** Mix proportions and raw material quantities of shield muck-based grouting material (kg).

Sample No.	Cement	RHA	Shield Muck	Water	Superplasticizer	Quicklime	HSCA
R0W2.4M	1.5	0	7.5	3.6	0.009	0	0
R15W2.4M	1.275	0.225	7.5	3.6	0.009	0	0
R20W2.4M	1.2	0.3	7.5	3.6	0.009	0	0
R25W2.4M	1.125	0.375	7.5	3.6	0.009	0	0
R20W2.4S	1.2	0.3	7.5	3.6	0.009	0	0
R20W2.4L	1.2	0.3	7.5	3.6	0.009	0	0
R25W2.4S	1.125	0.375	7.5	3.6	0.009	0	0
R25W2.4L	1.125	0.375	7.5	3.6	0.009	0	0
R25W2.5L	1.125	0.375	7.5	3.75	0.009	0	0
R25W2.6L	1.125	0.375	7.5	3.9	0.009	0	0
R25W2.6LC3	1.125	0.375	7.5	3.9	0.009	0.387	0
R25W2.6LC4	1.125	0.375	7.5	3.9	0.009	0.516	0
R25W2.6LC5	1.125	0.375	7.5	3.9	0.009	0.645	0
R25W2.6LC6	1.125	0.375	7.5	3.9	0.009	0.774	0
R25W2.6LC7	1.125	0.375	7.5	3.9	0.009	0.903	0
R25W2.6LH3	1.125	0.375	7.5	3.9	0.009	0	0.387
R25W2.6LH4	1.125	0.375	7.5	3.9	0.009	0	0.516
R25W2.6LH5	1.125	0.375	7.5	3.9	0.009	0	0.645
R25W2.6LH6	1.125	0.375	7.5	3.9	0.009	0	0.774
R25W2.6LH7	1.125	0.375	7.5	3.9	0.009	0	0.903

Note: “R” represents the RHA substitution rate; “W” represents the water-to-binder ratio; “S”, “M”, and “L” represent shield muck with small, medium, and large particle sizes, respectively; “C” represents quicklime; and “H” represents HSCA. For example, “R25W2.6LC3” denotes a mixture containing an RHA substitution rate of 25%, a water-to-binder ratio of 2.6, large particle size shield muck, and a quicklime dosage of 3%.

**Table 4:** Standards and normative references for the test methods.

Test	Standard Followed	International Reference
Consistency	JGJ/T 70-2009 [27]	EN 1015-3 [28]
Setting time	JGJ/T 70-2009 [27]	ASTM C403 [29]
Bleeding rate	T/CECS 563-2018 [30]	EN 480-4 [31]
Compressive strength	JGJ/T 233-2011 [32]	/

## Data Availability

The original contributions presented in this study are included in the article. Further inquiries can be directed to the corresponding author.
